# Seasonal variations in presenting symptoms and signs of dry eye disease in Norway

**DOI:** 10.1038/s41598-022-25557-9

**Published:** 2022-12-06

**Authors:** Jon Roger Eidet, Xiangjun Chen, Sten Ræder, Reza A. Badian, Tor P. Utheim

**Affiliations:** 1 The Norwegian Dry Eye Clinic, Oslo, Norway; 2 Department of Ophthalmology, Oslo University Hospital, Nydalen, P.O. box 4956, 0424 Oslo, Norway; 3 Department of Ophthalmology, Hospital of Southern Norway, Arendal, Norway; 4 Department of Medical Biochemistry, Oslo University Hospital, Oslo, Norway; 5 Department of Ophthalmology, Stavanger University Hospital, Oslo, Norway; 6 Department of Ophthalmology, Vestre Viken Hospital Trust, Drammen, Norway; 7 Department of Research and Development, Oslo Metropolitan University, Oslo, Norway; 8 Department of Clinical Medicine, Faculty of Medicine, University of Bergen, Bergen, Norway; 9 Department of Quality and Health Technology, The Faculty of Health Sciences, University of Stavanger, Stavanger, Norway; 10 Department of Oral Biology, Faculty of Dentistry, University of Oslo, Oslo, Norway; 11 National Centre for Optics, Vision and Eye Care, Department of Optometry, Radiography and Lighting Design, Faculty of Health Sciences, University of South-Eastern Norway, Kongsberg, Norway; 12 Department of Health and Nursing Science, The Faculty of Health and Sport Sciences, University of Agder, Grimstad, Norway

**Keywords:** Health care, Physical examination

## Abstract

The study investigated the seasonal variations of presenting symptoms and signs of dry eye disease (DED) in Norway. 652 consecutive DED patients examined between August 2012 and May 2015 in Oslo, Norway, were included. Presenting symptoms and signs were related to the season according to when each patient was examined. Weather report data from the examination day were compared with the presenting symptoms and signs. Oslo's mean seasonal temperatures during spring, summer, fall, and winter were 6.4 °C, 15.6 °C, 9.3 °C, and − 2.1 °C, respectively. Dry eye severity level and self-reported symptoms measured by the Ocular surface disease index questionnaire did not differ between seasons. Schirmer I was lower during summer than in other seasons (P < 0.01). The percentage of patients with a pathological tear meniscus height (< 0.2 mm) was higher during fall (P < 0.01) and lower during winter (P < 0.05) compared to the other seasons. Signs and symptoms of DED generally did not correlate with weather report data, although intraocular pressure was weakly associated with mean daily air temperature (r = − 0.22; P < 0.001). Neither dry eye severity level nor dry eye symptoms differ between seasons in Oslo, Norway. However, some parameters for assessing DED show seasonal variations (Schirmer I and tear meniscus height), which are essential to consider when examining patients with DED.

## Introduction

The hallmark of dry eye disease (DED) is hyperosmolar tears due to tear evaporation^1^, which initiates ocular surface damage and inflammation. The pathogenesis includes a cascade of events that leads to goblet cell/epithelial cell damage, loss of glycocalyx, and the recruitment of T-lymphocytes^1^. The prevalence of DED is estimated to range between 5 and 50%, depending on the populations studied and criteria used, and increases with age and female sex^2^. Dry eye symptoms in terms of burning, dry, gritty, itching, irritated, sandy, and tired eyes are among the most commonly reported complaints in the eye clinic^3^. The two main forms of DED are the aqueous deficiency and the evaporative type^1^. Aqueous deficiency can be seen in Sjögren’s syndrome and after radiation therapy to the lacrimal glands. Causes of evaporative DED are either endogenous, i.e., meibomian gland dysfunction (MGD), or external, i.e., environmental factors. Environmental causes of dry eyes consist of low humidity, wind, air pollution, and allergens^4,5^. It has been proposed that DED patients are susceptible to environmental changes^6^.

As a range of environmental factors can aggravate DED, it is expected that patients with DED show seasonal variation in their severity of symptoms and signs of dry eyes. In a large multicenter study by Kumar and co-workers that included 3.4 million consultations across the continental United States (US), the prevalence of dry eyes in US veterans was highest during winter/spring and lowest during summer^7^. The highest monthly prevalence was seen during April. Also, in a study from the Greater Boston Area, U.S., the severity of symptoms of dry eyes was found to be highest during winter^8^. In Bologna, Italy, symptoms of ocular discomfort were reported to be highest between November and February and between June and August^5^. Based on Google trend data in the United States, Canada, United Kingdom, and Australia, dry eyes searches on the internet also showed a slight seasonal variation with the highest incidence during summer^9^. In a multicenter study in Japan, on the other hand, no seasonal variation was found in the prevalence of DED^6^. A Danish study also reported a lack of variation in the prevalence of dry eyes, as indicated by reduced break-up time and increased lissamine green staining during winter compared to summer^10^. Thus, there are conflicting reports concerning seasonal variation in DED.

The current study represents the largest single-center study on seasonal variation of DED severity. It includes 652 patients examined at the Norwegian Dry Eye Clinic in Oslo between August 2012 and May 2015. Furthermore, it is the first to investigate if MGD, the most common cause of DED, displays seasonal variation.

## Results

### Patient demographics

Patient demographics are shown in Table 1.

**Table 1:** Study demographics.

	Spring	Summer	Fall	Winter	Sum/mean	*P*-value
Number of patients	216	52	167	217	652	–
Age (years)	52.3 ± 16.9	53.9 ± 16.4	51.5 ± 17.1	51.5 ± 16.5	52.0 ± 16.8 (range 8–91)	0.80^a^
Female/male	124/92	13/39^b^	87/80	143/74^c^	367/285	< 0.001^c^

Spring: March, April, and May; Summer: June, July, and August; Fall: September, October, and November; Winter: December, January, and February; ^a^One-way analysis of variance; ^b^a higher proportion of male patients than expected (P < 0.001; Chi-square test); ^c^Chi-square test.

### Seasonal weather data

Weather report data from Oslo are summarized in Table 2.

**Table 2:** Seasonal weather report data from Oslo between 2012 and 2015.

	Spring	Summer	Fall	Winter	P-value
Air temperature (°C)	6.4 ± 5.1	15.5 ± 1.8^b^	6.9 ± 4.6	− 2.3 ± 4.1^b^	< 0.001^a^
Relative air humidity (%)	68.1 ± 16.9^c^	68.7 ± 12.4	83.8 ± 8.0^d^	84.3 ± 9.6	< 0.001^a^
Hours of sunshine	5.9 ± 4.6	8.4 ± 5.0^e^	5.1 ± 3.8	1.6 ± 2.4^e^	< 0.001^a^
Wind speed (m/s)	2.9 ± 1.0	2.5 ± 0.5	3.0 ± 1.8f.	2.5 ± 1.3^ g^	< 0.001^a^

^a^One-way analysis of variance; ^b^*P* < 0.001 compared to all other seasons (Tukey post hoc test); ^c^*P* < 0.001 compared to all seasons except Spring (Tukey post hoc test); ^d^*P* < 0.001 compared to all seasons except Winter (Tukey post hoc test); ^e^*P* < 0.001 compared to all other seasons (Tukey post hoc test); ^f^*P* < 0.05 compared to Summer and Winter; ^g^*P* < 0.01 compared to Spring and Fall (Tukey post hoc test).

### Correlations between weather report data and clinical tests

Weather report data correlated the strongest with intraocular pressure, which was weakly associated with mean air temperature (C°) (*r* = − 0.22; *P* < 0.001), and wind speed (*r* = − 0.14; *P* < 0.015) (Table 3). Tear meniscus height correlated weakly with mean air temperature (*r* = − 0.12; *P* = 0.004). Dry eye severity level was marginally associated with relative air humidity (%) (*r* = 0.09; *P* = 0.032). Symptoms of DED as measured by the self-reported OSDI were not associated with any of the weather report data parameters.

**Table 3:** Correlations between weather report data and symptoms and signs of DED.

	Air temperature (C°)		Relative air humidity (%)		Hours of sunshine		Wind speed (m/s)	
	*r*	*P*-value	*r*	*P*-value	*r*	*P*-value	*r*	*P*-value
Ocular surface disease index	− 0.012	0.771	0.027	0.523	− 0.041	0.360	− 0.070	0.090
Dry eye severity level	0.016	0.684	**0.085**	**0.032**	− 0.076	0.084	− 0.048	0.231
Tear film breakup time	− 0.013	0.750	− 0.016	0.687	− 0.030	0.493	0.039	0.326
Ocular protection index	0.035	0.389	− 0.009	0.824	0.016	0.717	− 0.017	0.677
Schirmer I	− 0.048	0.229	− 0.021	0.601	0.031	0.481	0.022	0.582
Tear meniscus height (% < 0.2 mm)	− **0.115**	**0.004**	− 0.039	0.334	− 0.020	0.653	0.019	0.632
Ocular surface staining	− 0.014	0.720	0.029	0.457	− 0.049	0.267	− 0.014	0.727
Meibum expressibility	0.051	0.201	0.039	0.326	− 0.042	0.346	− 0.012	0.768
Intraocular pressure	− **0.222**	**< 0.001**	− 0.016	0.785	− 0.039	0.551	− **0.141**	**0.015**

*r* Pearson correlation, *DED* dry eye disease; Bold font = statistically significant.

### Symptoms and signs of dry eye disease according to season

Neither self-reported symptom load (OSDI) nor dry eye severity level (DESL) differed between seasons (Table 4). Schirmer I was lower during summer than during all other seasons (*P* < 0.01). The percentage of patients with tear meniscus height < 0.2 mm was higher during fall (29%) compared to other seasons (*P* < 0.001). Intraocular pressure was higher during winter than during summer and fall (*P* < 0.05).

**Table 4:** Ocular symptoms and signs according to season.

	Spring	Summer	Fall	Winter	P-value
Ocular surface disease index	36.7 ± 22.3	31.6 ± 19.4	35.4 ± 20.4	35.1 ± 21.5	0.51^a^
Dry eye severity level	2.0 ± 0.4	2.1 ± 0.5	2.0 ± 0.5	2.0 ± 0.5	0.85^a^
Dry eye severity level 1 (% within season)	8.5	10.2	10.2	10	–
Dry eye severity level 2 (% within season)	81.5	71.4	75.8	75.4	–
Dry eye severity level 3 (% within season)	9.5	18.4	14	14.7	–
Dry eye severity level 4 (% within season)	0.5	0	0	0	–
Tear film breakup time	5.0 ± 3.9	6.7 ± 10.1	5.6 ± 3.9	4.9 ± 3.9	0.34^a^
Ocular protection index	2.1 ± 2.2	2.7 ± 2.6	2.0 ± 1.6	1.8 ± 1.7	0.06^a^
Ocular surface staining	1.5 ± 2.0	1.6 ± 2.2	1.5 ± 2.0	1.7 ± 2.1	0.67^a^
Schirmer I	16.1 ± 10.5	10.1 ± 7.5^b^	15.8 ± 10.3	15.4 ± 10.3	**0.002**^**a**^
Tear meniscus height (% < 0.2 mm)	11.9	16.0	29.2^d^	8.7	**< 0.001**^**c**^
Meibum expressibility	1.1 ± 0.9	0.7 ± 0.8	1.0 ± 0.9	1.0 ± 0.9	0.051^a^
Intraocular pressure	13.9 ± 3.2	12.9 ± 2.4	13.1 ± 3.6	15.1 ± 3.7^e^	**< 0.001**^**a**^

^a^One-way analysis of variance; ^b^*P* < 0.01 compared to all other seasons (Tukey post hoc test); ^c^Chi-square test; ^d^higher percentage than expected (*P* < 0.001; Chi-square test); ^e^*P* < 0.05 compared to summer and fall (Tukey post hoc test); Bold font = statistically significant.

## Discussion

Understanding factors that are associated with DED is important for accurate diagnosis, and optimal treatment, of dry eye. Environmental factors such as high altitude^11,12^, exposure to wind^13^, air pollution^13,14^, and low relative humidity^15^ have been suggested to impact the DED. The current study was performed to assess whether parameters used in clinical DES diagnosis have a seasonal pattern. We hypothesized that seasonal environmental changes in temperature and humidity affect clinical parameters.

In the current study, DESL did not vary according to season. This is in line with two studies from Japan and Denmark, where no seasonal variation in the prevalence of DED could be found^6,10^. The lack of a consistent seasonal variation in DED could be related to a geographical impact. In the US, the prevalence of DED appeared higher in the southern regions^16^. In our study, all patients were examined at a single location. Hence no conclusions on regional effects can be drawn. Although the prevalence of DED was not investigated in our study, an increased prevalence of DED would be expected to be associated with a higher mean DESL. A large multicenter study that included 3.4 million consultations across the continental United States, on the other hand, did show a higher prevalence of dry eyes in US veterans during winter/spring than during summer^7^. The authors attributed the higher prevalence to be related to allergen exposure. Unfortunately, data on air allergen levels were not available for comparison in the present study. Dry eye severity level was only weakly related to higher specific humidity and was not associated with mean air temperature, average wind speed, or daily hours of sunshine in our study. The lack of associations between DESL, seasons, and weather report data in our study could be partly explained by the fact that the majority of the patients were classified as having DESL 2 (78%, data not shown). DESL is scored using a four-level system, thus, potential minute variations in DED severity may not have been detected with this scoring system.

In our study, self-reported symptoms of dry eyes, as measured by OSDI, did not show significant differences throughout the four seasons or with any single one of the weather report parameters. In the Greater Boston area, symptoms of dry eyes worsened during winter, which the authors speculated was related to more windy conditions, lower humidity, and lower air temperature^8^. A survey-based study in Sweden demonstrated that 47% of patients suffering from dry eye and/or Sjögren’s syndrome reported that they had the impression that seasonal conditions had a high impact on their DED symptoms, compared to only 15% reporting no presumed seasonal impact^17^. Wind (71%), followed by sunshine (60%) and heat (42%), were the most common weather conditions to reportedly impact on dry eye symptoms. In addition, cold weather also was suggested to aggravate DED symptoms in 34% of patients. The general lack of moderate/strong associations between symptoms of DED and weather conditions in our study, may, however, indicate that these environmental factors do not affect symptoms of DED to any large extent. Other factors, including allergen exposure and air pollution, were not assessed in this study but could represent relatively more important contributions to DED symptoms.

Signs of DED did not show a uniform variation throughout the various seasons in this study. Schirmer I and tear meniscus height were the only DED signs that were significantly correlated with season or weather report data. Schirmer I was lower during Summer compared to the remaining year. The known induction of tear secretion through the activation of corneal cold thermoreceptors could support this finding^18^. We also found a more pathological tear meniscus height during fall, a season during which relative air humidity is high in Oslo. Hence, this result is surprising given that tear evaporation is inversely related to relative air humidity^19^. Thus, there must be other aggravating factors that reduce tear meniscus height during the fall season in Oslo. A reduced ambient temperature during fall could increase meibomian lipid viscosity and inhibit meibomian gland secretion, thereby promoting tear evaporation due to a thinner tear lipid layer.

Intraocular pressure has been shown to increase during winter in normotensive individuals^20^ and in patients with ocular hypertension^21,22^. The approximately 2 mmHg increase in IOP during winter compared to summer in the current study is similar to what was shown in other studies on normotensive patients^20,23^. The mechanism of higher IOP during winter has been hypothesized to be related to the pineal hormone melatonin, as its secretion is inhibited by light and therefore reduced during summer^21^.

Surprisingly, meibum expressibility was not associated with the current season or air temperature in our study. This was expected since meibomian lipid viscosity, and its expressibility is related to temperature^24^. Other reports have shown higher meibum secretion following increased eyelid temperature by application of warm compresses. Apparently, warm compresses reduce secrete stagnation by offering a short-term decrease in lipid viscosity. As meibum expression was not reduced during winter, other factors may counteract the effect of temperature on lipid viscosity. Consumption of dietary omega-3 fatty acids has been demonstrated to improve symptoms of MGD^25^. Proposed mechanisms include the reduction of inflammation and alteration of the meibomian lipid composition^25^. A study from Belgium demonstrated lower serum levels of omega-3 fatty acids during winter than summer^26^, which would be expected to exacerbate MGD during winter. To our knowledge, the variation of serum level of omega-3 fatty acids throughout the year in a Norwegian population has not been reported. Finally, the ambient temperature in the offices of the Norwegian Dry Eye Clinic was not measured. However, it is reasonable to assume that it stayed relatively constant throughout the year. Still, the indoor temperature may be somewhat more important than the outdoor temperature regarding meibum expression. The latter may be significant to consider during the colder seasons in Norway, during which people tend to stay indoors.

In conclusion, there was no apparent seasonal variation in the level of dry eye severity or self-reported symptoms of dry eyes in patients being examined in Oslo, Norway. Certain parameters for assessing DED, on the other hand, did show seasonal variations that should be considered when diagnosing DED. Further studies including parameters on the indoor environment, as well as allergens and air pollution, could shed more light on the magnitude of external influences on DED.

## Methods

### Patients

The study was conducted in accordance with the Declaration of Helsinki. Written informed consent was obtained from all patients. The Regional Committee for Medical & Health Research Ethics, Section C, South East Norway (REC) reviewed the use of the data for the study from the Norwegian Dry Eye Clinic. REC determined that the research project “Evaluation of data from the Norwegian Dry Eye Clinic” is outside the remit of the Act on Medical and Health Research (2008) and therefore can be conducted without its approval. A letter of exemption by REC is provided. The study consecutively included patients with dry eye symptoms that were examined for the first time at the Norwegian Dry Eye Disease Clinic in Oslo, Norway, between August 2012 and May 2015. Patients without symptoms of dry eye were excluded.

### Ophthalmological examination

All included subjects were examined by one of two ophthalmologists at the clinic and completed a self-report questionnaire on symptoms of ocular dryness (Ocular Surface Disease Index [OSDI]). The OSDI questionnaire includes 12 items on symptoms of ocular dryness, as reported elsewhere^27^. All examinations were performed between 9 a.m. and 4 p.m. The ophthalmological examination consisted of measuring tear film break-up time (TFBUT)^28,29^, ocular staining (lissamine green and fluorescein)^30^, meibum expressibility^31^, Schirmer I^28^, tear meniscus height, and intraocular pressure measured with iCare tonometer. The ocular protection index (OPI)^29^ and dry eye severity level (DESL) (Table 5)^32^ were evaluated based on the measurements. In this study, meibomian gland dysfunction was defined as having a pathological score (> 0) on meibum expressibility. Only scores from the right eye of each subject were used for analyses.

**Table 5:** Ophthalmological work-up for dry eye disease.

Parameter	Scoring method	Pathological score
Dry eye severity level	Four-level composite score based on ocular discomfort, visual disturbance, conjunctival injection, conjunctival/corneal staining, other signs of corneal/tear pathology, signs of lid/meibomian gland pathology, TFBUT and Schirmer I	> 0
Ocular surface disease index	12 items on symptoms of ocular dryness (range: 0–100)	> 12
Tear film break-up time (TFBUT)	The interval in seconds between the last complete blink and the first appearance of a dry spot, or disruption in the tear film following instillation of fluorescein	≤ 10
Ocular protection index	TFBUT divided by the interblink interval (mean time in seconds between two complete blinks)	< 1
Schirmer I	Paper test strips are inserted in the lower lateral third of the conjunctival sac and the eyes are closed for 5 min. The wetting of the paper strip is then measured in millimeters	≤ 10
Ocular surface staining	Following fluorescein instillation, the staining scores of the exposed cornea and interpalpebral conjunctiva are summarized using the Oxford grading scheme (range: 0–15)	> 0
Meibum expressibility	Five glands in the lower lid are evaluated according to the number of expressible glands: 0, all glands; 1, three to four glands; 2, one to two glands; and 3, no glands (score range: 0–3)	> 0
Tear meniscus height	Measured in mm on the lower eye lid with a slit lamp microscope	< 0.2

### Meteorological data

Presenting symptoms and signs in each patient were related to the mean weather report data from Oslo during the season in which the patient had been examined. Spring was defined as March, April, and May, summer as June, July, and August, fall as September, October, and November, and winter as December, January, and February. The weather report data were obtained from the Norwegian Meteorological Institute and included air temperature, relative air humidity, hours of sunshine, and wind speed. In addition, weather report data from the exact day of examination in Oslo were correlated with the presenting symptoms and signs.

### Statistical analyses

Data are presented as mean ± standard deviation (SD). One-way analysis of variance with Tukey post hoc test (continuous variables) and chi-square test (dichotomous variables) was used for comparing symptoms and signs of DED during any one season with the rest of the year. Pearson’s correlation coefficient (*r*) was used for correlating weather report data with symptoms and signs of DED. *P* < 0.05 was considered significant (SPSS ver. 21.0).

## Data Availability

The datasets generated during and/or analysed during the current study are available from the corresponding author on reasonable request.

## Abbreviations

DED: Dry eye disease
MGD: Meibomian gland dysfunction
REC: The Regional Committee for Medical & Health Research Ethics, Section C, South East Norway
OSDI: Ocular surface disease index
TFBUT: Tear film break-up time
OPI: Ocular protection index
DESL: Dry eye severity level

## References

[1] Bron AJ (2017). TFOS DEWS II pathophysiology report. Ocul. Surf..

[2] Stapleton F (2017). TFOS DEWS II epidemiology report. Ocul. Surf..

[3] O'Brien PD, Collum LM (2004). Dry eye: Diagnosis and current treatment strategies. Curr. Allergy Asthma Rep..

[4] McCulley JP, Uchiyama E, Aronowicz JD, Butovich IA (2006). Impact of evaporation on aqueous tear loss. Trans. Am. Ophthalmol. Soc..

[5] Versura P, Profazio V, Cellini M, Torreggiani A, Caramazza R (1999). Eye discomfort and air pollution. Ophthalmologica.

[6] Hikichi T (1995). Prevalence of dry eye in Japanese eye centers. Graefes Arch. Clin. Exp. Ophthalmol..

[7] Kumar N, Feuer W, Lanza NL, Galor A (2015). Seasonal variation in dry eye. Ophthalmology.

[8] Davis, J. A. *et al.* In *ARVO*, vol. 47 (Investigative Ophthalmology & Visual Science, 2006).

[9] Leffler CT, Davenport B, Chan D (2010). Frequency and seasonal variation of ophthalmology-related internet searches. Can. J. Ophthalmol..

[10] Franck C, Palmvang IB (1993). Break-up time and lissamine green epithelial damage in 'office eye syndrome'. Six-month and one-year follow-up investigations. Acta Ophthalmol. (Copenh.).

[11] Lu P (2008). Dry eye syndrome in elderly Tibetans at high altitude: A population-based study in China. Cornea.

[12] Willmann G (2014). Exposure to high altitude alters tear film osmolarity and breakup time. High Alt. Med. Biol..

[13] Sahai A, Malik P (2005). Dry eye: Prevalence and attributable risk factors in a hospital-based population. Indian J. Ophthalmol..

[14] Torricelli AA (2013). Correlation between signs and symptoms of ocular surface dysfunction and tear osmolarity with ambient levels of air pollution in a large metropolitan area. Cornea.

[15] Abusharha AA, Pearce EI (2013). The effect of low humidity on the human tear film. Cornea.

[16] McCann P (2022). Prevalence and incidence of dry eye and meibomian gland dysfunction in the United States: A systematic review and meta-analysis. JAMA Ophthalmol..

[17] van Setten G, Labetoulle M, Baudouin C, Rolando M (2016). Evidence of seasonality and effects of psychrometry in dry eye disease. Acta Ophthalmol..

[18] Parra A (2010). Ocular surface wetness is regulated by TRPM8-dependent cold thermoreceptors of the cornea. Nat. Med..

[19] Uchiyama E, Aronowicz JD, Butovich IA, McCulley JP (2007). Increased evaporative rates in laboratory testing conditions simulating airplane cabin relative humidity: An important factor for dry eye syndrome. Eye Contact Lens.

[20] Blumenthal M, Blumenthal R, Peritz E, Best M (1970). Seasonal variation in intraocular pressure. Am. J. Ophthalmol..

[21] Qureshi IA (1999). Seasonal and diurnal variations of ocular pressure in ocular hypertensive subjects in Pakistan. Singap. Med. J..

[22] Gardiner SK, Demirel S, Gordon MO, Kass MA, Ocular Hypertension Treatment Study, G. (2013). Seasonal changes in visual field sensitivity and intraocular pressure in the ocular hypertension treatment study. Ophthalmology.

[23] Bengtsson B (1972). Some factors affecting the distribution of intraocular pressures in a population. Acta Ophthalmol. (Copenh.).

[24] Butovich IA, Arciniega JC, Wojtowicz JC (2010). Meibomian lipid films and the impact of temperature. Investig. Ophthalmol. Vis. Sci..

[25] Thode AR, Latkany RA (2015). Current and emerging therapeutic strategies for the treatment of meibomian gland dysfunction (MGD). Drugs.

[26] De Vriese SR, Christophe AB, Maes M (2004). In humans, the seasonal variation in poly-unsaturated fatty acids is related to the seasonal variation in violent suicide and serotonergic markers of violent suicide. Prostaglandins Leukot. Essent. Fatty Acids.

[27] Schiffman RM, Christianson MD, Jacobsen G, Hirsch JD, Reis BL (2000). Reliability and validity of the Ocular Surface Disease Index. Arch. Ophthalmol..

[28] Bron AJ (2001). Diagnosis of dry eye. Surv. Ophthalmol..

[29] Ousler GW, Hagberg KW, Schindelar M, Welch D, Abelson MB (2008). The ocular protection index. Cornea.

[30] Bron AJ, Evans VE, Smith JA (2003). Grading of corneal and conjunctival staining in the context of other dry eye tests. Cornea.

[31] Nichols KK (2011). The international workshop on meibomian gland dysfunction: Executive summary. Investig. Ophthalmol. Vis. Sci..

[32] The Definition and Classification of Dry Eye Disease (2007). Report of the definition and classification subcommittee of the international dry eye workshop (2007). Ocul. Surf..

